# Helminth and ectoparasitic faunas of the Harris’s hawk, *Parabuteo unicinctus* (Accipitriformes: Accipitridae), in Chile: new data on host-parasite associations for Neotropical raptors

**DOI:** 10.1590/S1984-29612022046

**Published:** 2022-08-19

**Authors:** Pablo Oyarzún-Ruiz, Camila Cifuentes-Castro, Félix Varas, Alexandra Grandón-Ojeda, Armando Cicchino, Sergey Mironov, Lucila Moreno

**Affiliations:** 1 Facultad de Ciencias de la Naturaleza, Universidad San Sebastián, Concepción, Chile; 2 Departamento de Zoología, Facultad de Ciencias Naturales y Oceanográficas, Universidad de Concepción, Concepción, Chile; 3 Laboratorio de Parásitos y Enfermedades en Fauna Silvestre, Facultad de Ciencias Veterinarias, Universidad de Concepción, Chillán, Chile; 4 School of Biological Sciences, University of Bristol, Bristol, United Kingdom; 5 Universidad Nacional de Mar del Plata, Mar del Plata, Argentina; 6 Zoological Institute, Russian Academy of Sciences, Universitetskaya Embankment 1, Saint Petersburg, Russia

**Keywords:** Birds of prey, Accipitriformes, parasites, neotropics, Chile, Aves de rapina, Accipitriformes, parasitas, neotrópico, Chile

## Abstract

Birds of prey harbor a wide spectrum of various parasites, mostly with a heteroxenous life cycle. However, most reports on their parasites come from Europe. Although the Harris’s hawk (*Parabuteo unicinctus*) is a widespread species in America, parasitological surveys on this hawk are mostly focused on coprological findings and ectoparasites, with poor attention paid to helminths. The aim of this study was to gather new and additional data on host-parasite associations for the Harris’s hawk. Twenty-nine birds from central and southern Chile were necropsied. Further, nine birds from a rehabilitation center and 22 museum specimens were inspected for ectoparasites. Sixty-eight percent of birds hosted at least one parasite species. Four lice species, one mite species and eight helminth species (five nematodes, two platyhelminthes and one acanthocephalan) were recorded. Parasitic lice *Colpocephalum nanum* and *Nosopon chanabense*, and a nematode *Cyathostoma* (*Hovorkonema*) *americana* were recorded for the first time in raptors from the Neotropics. A feather mite, *Pseudalloptinus* sp., nematodes, *Physaloptera alata* and *Microtetrameres* sp., and a trematode *Neodiplostomim travassosi*, were recorded for the first time in Chile. The presence of diverse heteroxenous helminths reported here in the Harris’s hawk could be explained by the generalist diet of this raptor.

## Resumo

As aves de rapina representam uma fonte importante de parasitas heteroxenos. Porém, a maioria dos relatos são da Europa. O gavião-asa-de-telha (*Parabuteo unicinctus*) é uma espécie amplamente distribuída no continente Americano, porém estudos parasitológicos nessas aves têm documentado achados coprológicos e ectoparasitas, com poucos relatos sobre endoparasitas como helmintos. Por essa razão, o objetivo deste estudo foi descrever novas associações parasita-hospedeiro para o gavião-asa-de-telha e rapinantes neotropicais. Vinte nove aves provenientes do centro e sul do Chile foram submetidas à necropsia. Por outro lado, nove aves de um centro de reabilitação e 22 espécimes de museu foram inspecionados em busca de ectoparasitas. Do total de aves, 68,3% foram identificadas como portadoras de pelo menos uma espécie de parasita. Quatro espécies de piolhos, um ácaro e oito helmintos (cinco nematoides, dois platelmintos e um acantocéfalo) foram registrados. Os piolhos *Colpocephalum nanum*, *Nosopon chanabense* e o nematóide *Cyathostoma* (*Hovorkonema*) *americana* são reportados pela primeira vez em rapinantes neotropicais. *Colpocephalum nanum, N*. *chanabense*, *Pseudalloptinus* sp., *Physaloptera alata*, *Microtetrameres* sp., *C*. (*H*.) *americana* e *Neodiplostomim travassosi,* são reportados pela primeira vez no Chile. A diversidade de helmintos heteróxenos, identificados neste trabalho, poderia ser explicada devido à dieta geralista do gavião-asa-de-telha.

## Introduction

The parasitic fauna of birds of prey is rich in species with direct and indirect life cycles. In most cases, birds of prey are the definitive hosts of helminths, acquiring them through the ingestion of intermediate and paratenic hosts, such as birds, micromammals, earthworms, and arthropods (Krone, 2000; Krone & Cooper, 2002; Atkinson et al., 2008). Despite the wide geographical distribution of birds of prey, most studies of their host-parasite associations have been carried out in European countries (Krone, 2000). Additionally, parasitism can be costly to the host, as pathological changes in tissues, immune response, and loss of nutrients may occur (Krone & Cooper, 2002; Atkinson et al., 2008). However, the possible effects of parasitism on the health of birds of prey is still largely unknown, as particular records have been identified in certain species (Lavoie et al., 1999; Krone & Cooper, 2002; Atkinson et al., 2008; Santoro et al., 2010; Vaughan-Higgins et al., 2013).

The Harris’s hawk *Parabuteo unicinctus* (Temminck, 1824) (Accipitriformes, Accipitridae) is a raptor that is widely distributed in the Americas with two recognized subspecies: *P*. *u*. *harrisi* (Audubon, 1837) in North America and part of South America; and *P*. *u*. *unicinctus* restricted to the Neotropics (Ferguson-Lees & Christie, 2005). This raptor is widely distributed in various environments occurring in forests, agricultural lands, and also in urbanized areas with patches of arboreal cover (Pavez, 2019).

There are some studies related to the parasitic fauna on the Harris’s hawks from the Neotropics, although most of these studies have been focused on coprological analyses (Santos et al., 2011, 2015; Santana-Sánchez et al., 2015) and ectoparasites (Clay, 1958; Cicchino & Castro, 1998a, b; Philips, 2000; González-Acuña et al., 2008), with a few reporting helminths (Dubois, 1937; Morgan, 1943; Yamaguti, 1961). Even though coprological analyses are inexpensive, they do not always allow specific diagnoses of endoparasites when compared to collections from parasitological necropsy (Lutz et al., 2017; Oyarzún-Ruiz & González-Acuña, 2020).

There are 35 species of birds of prey in Chile (Pavez, 2019), of which 23 have been recorded as hosts of some parasitic species. However, most of these studies focused on ectoparasites, with only 4 species reported as hosts of helminths (Moreno & González-Acuña, 2015; Oyarzún-Ruiz et al., 2016; Grandón-Ojeda et al., 2018, 2019). Additionally, parasite surveys of the Harris’s hawk in Chile previously documented only one record for lice (González-Acuña et al., 2008), with no data on mites or helminths. Thus, the aim of the present study was to detail the ectoparasitic and helminth fauna of the Harris’s hawk in Central and Southern Chile, establishing additional and new records for Neotropical raptors.

## Materials and Methods

**Figure 1:**
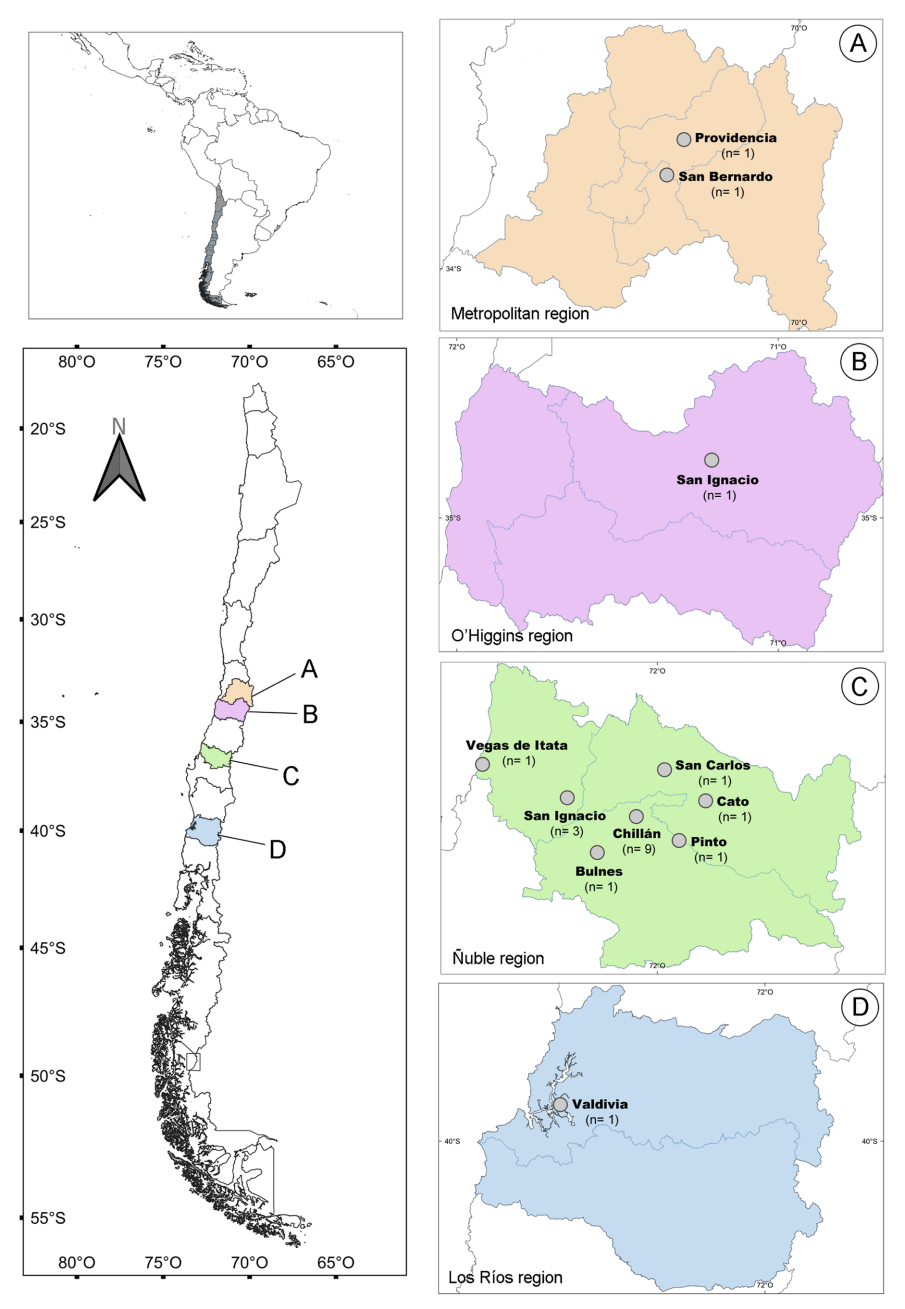
Map showing the locations from which the hawks originated. The number of necropsied hawks (n) for every location is detailed between parentheses.

A total of 29 Harris’s hawk carcasses from different localities (Figure 1) were necropsied from 2017 to 2020. Eight were obtained from the Metropolitan region (San Bernardo and Providencia commune, n=1 each; and with no locality recorded, n=6), three – from O’Higgins region (San Ignacio commune, n=1; and with no locality recorded, n=2), 17 – from Ñuble region (Chillán, n=9; Cato, n=1; San Ignacio, n=3; San Carlos, n=1; Pinto, n=1; Bulnes commune, n=1; and Vegas de Itata, n=1), and one – from Los Ríos region (Valdivia) (Figure 1). All of these birds were found dead or were euthanized in rehabilitation centers for humanitarian reasons. All birds were transported for parasitological examination to the Laboratorio de Parásitos y Enfermedades de Fauna Silvestre, Facultad de Ciencias Veterinarias, Universidad de Concepción, Chillán, Chile, where they were immediately necropsied or frozen until analyses were performed. No ethical committee’s approval was necessary for the use of these carcasses. The age of each bird was determined according to the chromatism of feathers (Pavez, 2019), and then confirmed through the presence/absence of the bursa of Fabricius during necropsy; the sex of each bird was determined via inspection of its gonads. Of these, 10 birds were males (2 immatures, 6 juveniles, 1 adult, and 1 with no age data), 12 were females (4 immatures, 7 juveniles, and 1 with no age data), and 7 had no age or sex data.

Additionally, between 2013 and 2017, nine Harris’s hawks were screened for ectoparasites and rehabilitated at the facilities of the Centro de Rehabilitación de Fauna Silvestre (CR), Universidad de Concepción, Chillán, Chile. The skins from 22 Harris’s hawks that had been deposited in the Museo Nacional de Historia Natural (MNHN), Santiago, Chile were included in the present study and inspected for ectoparasites.

The presence of ectoparasites was determined by inspecting feathers of the head, body, wings, and rectrices under a stereomicroscope. Then, using an insecticide based on pyrethrin (Cyperkill 25 EC), the “dust-ruffling” technique described by Walther & Clayton (1997) was performed to collect additional ectoparasites. To determine the presence of nasal mites, the modified Yunker’s method was performed (Wilson, 1964) in the necropsied birds, as achieved through nasal flushing using a water-soap solution and rinsed with tap water. Then, the nasal sinuses, nasal turbinates, orbital cavities, and nares were exposed and inspected under stereomicroscope. All collected ectoparasites were deposited in 70% ethanol.

The feather mites were cleared in the Nesbitt’s solution in a thermal bath (Dry Bath Incubator MK2000-1) at 70 °C for 10 minutes. Then, all mites were mounted onto slides containing the Berlese medium (Krantz & Walter, 2009). Collected lice were cleared with KOH 20% then dehydrated in a series of ethanol concentrations (40%, 80%, and 100%), cleared in clove oil for 24 hours, and mounted onto slides with Canada balsam (Palma, 1978; Price et al., 2003). The mites were identified using the descriptions by Gaud (1988). Lice were identified according to Clay (1958), Tendeiro (1959), Price & Beer (1963), and Price et al. (2003).

Parasitic necropsy was performed following the methods of Lutz et al. (2017); thus, the eyes, esophagus, gastrointestinal tract, trachea, lungs, heart, liver, gallbladder, kidneys, bursa of Fabricius, subcutaneous tissue, and articulations were examined under stereomicroscope. Every organ was dissected and/or crushed in citrated saline and then mixed using a bottle, followed by repetitive sedimentations. Once all organs were removed, the coelomic cavity was washed using the former solution. The sediment of every organ and cavity was examined under stereomicroscope. All collected helminths were relaxed in saline and preserved in 80% ethanol according to the methods of Lutz et al. (2017), and Oyarzún-Ruiz & González-Acuña (2020).

For the preparation of helminths, acanthocephalans were cleared in a temporary mounting medium with glycerin ethanol for at least 5 days. Meanwhile, tapeworms and flukes were stained using Alum carmine stain, dehydrated in a series of ethanol concentrations (70%, 80%, 96%, and 100%), cleared in clove oil, and mounted onto slides using Canada balsam (Lutz et al., 2017; Oyarzún-Ruiz & González-Acuña, 2020). Nematodes were identified following Cram (1927), Morgan & Schiller (1950), Yamaguti (1961), Baruš et al. (1978), Borgsteede & Okulewicz (2001), Anderson et al. (2009), and Kanarek et al. (2016); and acanthocephalans were identified following Yamaguti (1963) and Smales (2013). Meanwhile, tapeworms were identified according to Yamaguti (1959) and Khalil et al. (1994), and flukes following the keys detailed by Gibson et al. (2002), Lunaschi & Drago (2005), and Drago et al. (2014). Ectoparasites and helminths were examined using an optic microscope (Leica DM1000; Leica Microsystems, Wetzlar, Germany).

Prevalence (P), intensity (I), range (R), mean intensity (_M_I), and mean abundance (_M_A) were estimated and interpreted according to Bush et al. (1997).

All parasites were deposited in the parasitological collection of Laboratorio de Parásitos y Enfermedades de Fauna Silvestre, Universidad de Concepción.

## Results

Forty-one of the 60 inspected hawks (68.3%) were parasitized by at least one species of parasite; 17 out 29 necropsied hawks (17/29; 58.6%) were found parasitized by helminth parasites. Also, of the nine hawks screened at the CR, five were found to be parasitized: three birds with chewing lice, and the other two with feather mites. With respect to the skins deposited in the MNHN, 19 out of 22 skins were found with chewing lice and feather mites.

Regarding ectoparasites, the following lice species were recorded: *Degeeriella emersoni* (Clay, 1985) in three birds (33.3%), and *Craspedorrhynchus* sp. (Philopteridae) in one bird (11.1%), both of which were identified from the body feathers of captive hawks at CR. In addition to feather mites, *Pseudalloptinus* sp. (Pterolichidae), was also collected in two captive hawks at CR. Furthermore, from the deposited skins, two species of lice were recorded: *Colpocephalum nanum* Piaget, 1890 (Menoponidae) from 19 skins (86.4%) and *Nosopon chanabense* (Ansari, 1956) (Menoponidae) from three skins (13.6%) (Table 1).

**Table 1:** Parasites collected from the Harris’s hawk, *Parabuteo unicinctus*, from central and southern Chile (n=60).

**Parasite**	**n**	**P (%)**	**I**	**R**	**_M_I**	**_M_A**	**Site of collection**	**Location**
Phthiraptera								
Family Philopteridae								
*Degeeriella emersoni*	3	33.3	7	1-5	2.3	0.8	Body feathers	CR
*Craspedorrhynchus* sp.	1	11.1	22	22	22	2.4	Body feathers	CR
Family Menoponidae								
*Colpocephalum nanum*	19	86.4*	26	1-2	1.4*	1.2^*^	Body feathers	MNHN
*Nosopon chanabensis*	3	13.6*	4	1-2	1.3*	0.2*	Body feathers	MNHN
								
Acari								
Family Pterolichidae								
*Pseudalloptinus* sp.	2	22.2	92	1-62	46	10.2	Wing feathers	CR
								
Phylum Nematoda								
Family Ascarididae								
*Porrocaecum depressum*	1	3.5	1	1	1	0.03	Small intestine	Ni (RM)
Family Physalopteridae								
*Physaloptera alata*	2	6.9	9	1-8	4.5	0.3	Esophagus	Ni (VI); Chillán (XVI)
Family Tetrameridae								
*Microtetrameres* sp.	6	20.7	19	1-6	3.2	0.7	Proventriculus, small intestine	Providencia (RM); Ni (VI); San Ignacio (XVI); Valdivia (XIV)
Family Syngamidae								
*Cyathostoma* (*Hovorkonema*) *americana*	1	3.5	7	7	7	0.2	Air sacs, lungs	San Ignacio (XVI)
Family Capillariidae								
*Capillaria tenuissima*	4	13.8	4	1	1	0.1	Esophagus, small intestine, colon	Bulnes, Chillán (XVI); Valdivia (XIV)
								
Phylum Platyhelminthes								
Class Cestoda								
Family Paruterinidae								
*Cladotaenia* sp.	4	13.8	13	1-8	3.3	0.5	Small intestine, cloaca	Ni (VI); San Bernardo (RM); Chillán, San Ignacio (XVI)
Class Digenea								
Family Diplostomidae								
*Neodiplostomum travassosi*	4	13.8	47	1-26	11.8	1.6	Small intestine	Bulnes, Cato, San Ignacio (XVI)
								
Phylum Acanthocephala								
Family Centrorhynchidae								
*Centrorhynchus* sp.	1	3.5	1	1	1	0.03	Small intestine	Chillán (XVI)

Abbreviature: n= parasitized birds; P = prevalence; I = intensity of infection; R = range; _M_I= mean intensity; _M_A= mean abundance;*proportion estimated from the 22 skins from MNHN; Regions: RM = Metropolitan region; VI = O'Higgins region; XVI = Ñuble region; XIV = Los Ríos region.

For helminth parasites, 17 hawks were parasitized by at least one helminth species. A total of 101 worms from eight species of three phyla were collected. For Nematoda, the following species were recorded: *Porrocaecum depressum* (Zeder, 1800) Baylis, 1920 (Ascarididae) (3.5%) from the small intestine; *Physaloptera alata* Rudolphi, 1819 (Physalopteridae) (6.9%) from the esophagus; *Microtetrameres* sp. (Tetrameridae) (20.7%) from the proventriculus and small intestine; *Cyathostoma* (*Hovorkonema*) *americana* Chapin, 1925 (Syngamidae) (3.5%) from the aerial sacs and lungs and *Capillaria tenuissima* (Rudolphi, 1809) Yamaguti, 1941 (Capillariidae) (13.8%) mostly from the small intestine of birds. Also, two taxa of Platyhelminthes were collected: *Cladotaenia* sp. (Cestoda: Paruterinidae) (13.8%) and *Neodiplostomum travassosi* Dubois, 1937 (Trematoda: Diplostomidae) (13.8%), both of which were mostly collected from the small intestine. Finally, an unidentified species of acanthocephalan was isolated from the small intestine: *Centrorhynchus* sp. (Centrorhynchidae) (3.5%) (Table 1). Besides, 11 birds were parasitized by only one species of helminth, with six birds parasitized by two different species of helminths: *Microtetrameres* sp.+*N. travassosi*; *C. tenuissima*+*N. travassosi*; *Microtetrameres* sp.+*P. alata*; *Microtetrameres* sp.+*Cladotaenia* sp. (2 birds); and *Microtetrameres* sp.+*C. tenuissima*. The highest parasitic load was 26 worms, while the lowest was one worm (Table 1).

## Discussion

Almost 70% of the inspected hawks were parasitized by at least one taxon of parasite. A total of 13 taxa was collected: four chewing lice, one feather mite, five nematodes, two platyhelminths, and one acanthocephalan, with one helminth and three ectoparasites recorded for the first time in the Neotropics.

*Craspedorrhynchus* Kéler, 1938 and *Degeeriella* Neumann, 1906 are lice restricted to accipitrid (Accipitriformes) and falconid (Falconiformes) birds of prey (Price et al., 2003). In Chile, these genera have been recorded in the red-backed hawk [*Geranoaetus polyosoma* (Quoy & Gaimard, 1824)] (Accipitriformes) (Grandón-Ojeda et al., 2019). For the Harris’s hawk, there are previous records of *Craspedorrhynchus* sp. in Argentina (Cicchino & Castro, 1998a) and Chile (González-Acuña et al., 2008). The morphotype reported previously in Chile (see Moreno & González-Acuña, 2015) is the same collected in the present survey. Considering the restricted range of hosts for every louse species (Price et al., 2003), and given that the present louse is different to the other species, it probably corresponds to a new taxon, as Cicchino & Castro (1998a) suggested. Bearing in mind that only a few male specimens were collected, future revision of this material would clarify its taxonomic position. On the other hand, *D*. *emersoni* has a limited range of known hosts with only a few records in the Neotropics: *Buteogallus aequinoctialis* (Gmelin, 1788), *B. gundlachii* (Cabanis, 1855) (Accipitriformes) from Cuba (Clay, 1958; Price et al., 2003), and *P. unicinctus* from Argentina and an undetermined locality (Clay, 1958; Cicchino & Castro, 1998a). In Chile, this species has been recorded from the same host by González-Acuña et al. (2008). Furthermore, other species of *Degeeriella*, such *Degeeriella carruthi* (Emerson, 1955), *D. elani* Tendeiro, 1955, *D. epustulata* (Carriker, 1903), *D. fulva* (Giebel, 1874), *D. leucopleura* (Nitzsch, 1874), and *D. rufa* (Burmeister, 1838) have been reported infesting other raptors distributed across the country (Moreno & González-Acuña, 2015; Grandón-Ojeda et al., 2019).

*Colpocephalum* spp. parasitize several avian orders such Ciconiiformes, Columbiformes, Cuculiformes, Galliformes, Passeriformes, Pelecaniformes, Piciformes, Psittaciformes, Falconiformes, Accipitriformes, and Strigiformes (Price et al., 2003). *Colpocephalum nanum*, although with a widespread distribution, seems to be restricted to accipitrid raptors (Catanach et al., 2018), with records for *Accipiter gentilis* (Linnaeus, 1758), *A. cooperii* (Bonaparte, 1828), *A. nisus* (Linnaeus, 1758), *A. melanoleucus* Smith, 1830, *Buteo buteo* (Linnaeus, 1758), *B. lineatus* (Gmelin, 1788), *B. lagopus* (Pontoppidan, 1763), *B. rufinus* (Cretzschmar, 1829), *B*. *jamaicensis*, and *Circaetus cinereus* (Vieillot, 1818) from Africa, Asia, Europe, and North America (Price & Beer, 1963; Price et al., 2003). Thus, to the best of our knowledge, the current record is the first from a Neotropical bird of prey. The genus *Nosopon* Hopkins, 1950 has been recorded mostly from Holarctic raptors, including falconids and vultures (Tendeiro, 1959). *Nosopon chanabense* has been recorded only from accipitrid raptors such as *Aquila vindhiana* Franklin, 1831 and *Gyps himalayensis* Hume, 1869, both from India (Tendeiro, 1959). There is scarce knowledge related to this species in Neotropical birds of prey, with only one previous record infesting *Rosthramus sociabilis* (Vieillot, 1817) from Argentina (Cicchino & Castro, 1998b). The other species recorded from South America is *Nosopon lucidum* (Rudow, 1869) infesting *Falco sparverius* Linnaeus, 1758 (Falconiformes) from Argentina (Cicchino & Castro, 1998b). This finding represents an additional host-parasite association, as it is the second record to be identified in the Neotropics and the first for Chile.

**Figure 2:**
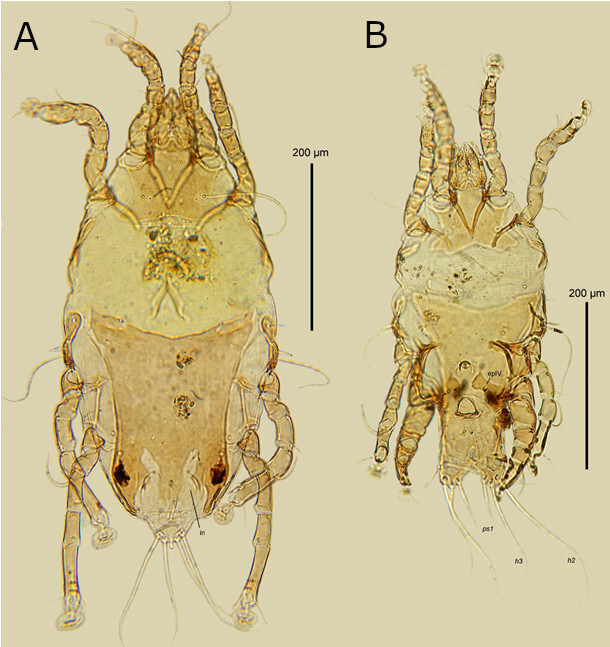
*Pseudalloptinus* sp. from Harris’s hawk. (A) female, dorsal view, in - incision in the posterior end of hysteronotal shield; (B) male, ventral view, *h2*, *h3*, *ps1* - terminal setae of opisthosomal lobes, epIV - epimerites IV.

Only one unidentified feather mite belonging to the genus *Pseudalloptinus* Dubinin, 1956 (Pteroichidae: Pterolichinae) was recorded. This genus currently includes six valid species, all restricted to birds of the order Accipitriformes; among them, *Pseudalloptinus aquilinus* (Trouessart, 1884) infesting *Aquila chrysaetos* (Linnaeus, 1758) and *Haliaeetus leucocephalus* (Linnaeus, 1766) was reported from the Holarctic and Paleotropic regions (Gaud & Mouchet, 1959; Gaud, 1988; Galloway et al., 2014; Mironov et al., 2018; Waki et al., 2021). For the Harris’s hawk, an unidentified *Pseudalloptinus* species was previously reported without exact locality, apparently from North America, by Philips (2000). To the best of our knowledge, our finding is the first record of this genus in the Neotropics. The *Pseudalloptinus* species collected from the Harris’s hawk in Chile is supposedly a new species and closest to *P. milvulinus* (Trouessart, 1884) and *P. africanus* Gaud, 1988 distributed on various accipitrids in the Old World. This mite differs from the two listed species in having setae *ps1* strongly thickened and epimerites IV widened in males and the hysteronotal shield with a pair of deep and narrow incisions in females (Figure 2).

*Porrocaecum depressum* is an ascarid worm that parasitizes the small intestine of birds of prey around the world (Morgan & Schiller, 1950; Yamaguti, 1961; Baruš et al., 1978; Lamothe-Argumedo et al., 1997; Borgsteede et al., 2003; Atkinson et al., 2008). Its life cycle is indirect, using earthworms as intermediate hosts and micromammals as paratenic hosts (Atkinson et al., 2008; Richardson & Kinsella, 2010). In Chile, it has been previously reported in *Milvago chimango* (Vieillot, 1816) (Falconiformes) (San Martín et al., 2006; Oyarzún-Ruiz et al., 2016). Thus, this finding states to Harris’s hawk as an additional host in South America.

*Physaloptera alata* has been recorded parasitizing birds of prey worldwide, serving as their definitive hosts and mainly inhabiting the esophagus and stomach of these birds (Pinto et al., 1994; Krone, 2000; Borgsteede et al., 2003; Sanmartín et al., 2004; Santoro et al., 2010). The life cycle of this parasite probably involves the participation of arthropods as intermediate hosts (Cram, 1927; Anderson, 2000). Only one unidentified *Physaloptera* has been recorded in Chile, which was isolated from *F*. *sparverius* in Central Chile (González-Acuña et al., 2011). Thus, this record represents an expansion in the geographical distribution of this nematode in the Neotropics (Schuurmans-Stekhoven, 1951; Pinto et al., 1994). *Physaloptera acuticauda* Molin, 1860 and *Physaloptera inflata* Molin, 1860 have been recorded in *P. unicinctus* from South America (Cram, 1927; Yamaguti, 1961).

*Microtetrameres* (Travassos, 1915) inhabits the proventriculus of birds, with females strictly associated with proventricular glands and males with the proventricular mucosa (Anderson, 2000; Díaz et al., 2018). Members of the family Tetrameridae have an indirect life cycle, with arthropods such as grasshoppers and cockroaches serving as intermediate hosts (Anderson, 2000). This genus has been recorded in raptors of orders Accipitriformes, Falconiformes, and Strigiformes worldwide (Illescas Gomez et al., 1993; Krone, 2000; Sanmartín et al., 2004; Honisch & Krone, 2008; Komorová et al., 2017). In South America, there are few records for this genus, with most of them coming from passerine birds and only one record from a bird of prey. Díaz et al. (2018) recently reported a new species for the Neotropics, *Microtetrameres urubitinga* Díaz, Drago & Núñez, 2018, parasitizing the proventriculus of *Buteogallus urubitinga* (Gmelin, 1788) in Argentina. The present *Microtetrameres* sp. is different from *M. urubitinga* and the other species reported for the Neotropics and North America; thus, it probably corresponds to a new taxon. Additional analysis will be performed to determine its specific taxonomic position. The present finding represents the geographical expansion of the distribution for this genus, considering that this is the first record for Chile (see Oyarzún-Ruiz & González-Acuña, 2021). Additionally, this record represents a new host-parasite association.

*Cyathostoma* (*H*.) *americana* has been reported from the trachea, lungs, and air sacs of birds of prey (Borgsteede & Okulewicz, 2001; Borgsteede et al., 2003; Kanarek et al., 2016). The species of this genus have a direct life cycle, although they could use earthworms as paratenic hosts (Anderson, 2000; Atkinson et al., 2008). These nematodes were found to cause severe pneumonia and pyogranulomatous air sacculitis, which are often associated with high parasitic loads (20-100 worms) (Lavoie et al., 1999; Atkinson et al., 2008; Vaughan-Higgins et al., 2013). However, in the present study, there were no macroscopic pathological changes associated with its presence, probably because of the low parasitic load. The present finding corresponds to the second record for Syngamidae found in a wild bird from Chile (see Oyarzún-Ruiz & González-Acuña, 2021); it also represents the first record of this species in a Neotropical bird of prey. Despite the low prevalence of these respiratory nematodes, as reported in previous studies (Lavoie et al., 1999; Borgsteede & Okulewicz, 2001; Borgsteede et al., 2003; Sanmartín et al., 2004) and as described in the present survey, there is a clear need for the prospection of extraintestinal parasites, considering that most of the studies performed thus far in the country have focused on gastrointestinal parasites (Oyarzún-Ruiz & González-Acuña, 2021). Furthermore, a similar situation occurs in other surveys from the Neotropics.

*Capillaria tenuissima* is a nematode with an unknown life cycle, although earthworms and rodents could act as intermediate and paratenic hosts, respectively (Atkinson et al., 2008). In the Neotropics, unidentified *Capillaria* eggs have been recorded from raptors such *Buteo swainsoni* Bonaparte, 1838, *Rupornis magnirostris* (Gmelin, 1788) (Accipitriformes), and *Bubo virginianus* (Gmelin, 1788) (Strigiformes) (Santos et al., 2011, 2015). In Chile, this nematode was previously recorded from *M. chimango* (San Martín et al., 2006; Oyarzún-Ruiz et al., 2016) and *Bubo magellanicus* (Gmelin, 1788) (Grandón-Ojeda et al., 2018). Other capillarids reported from raptors include the following genera: *Baruscapillaria* Moravec, 1982; *Eucoleus* Diyardin, 1845; and *Pterothominx* Freitas, 1959 (Borgsteede et al., 2003; Richardson & Kinsella, 2010; Santoro et al., 2010; González-Acuña et al., 2011; Oyarzún-Ruiz et al., 2016; Honisch & Krone, 2008). This species was not a prevalent nematode, as noted in previous studies (see Borgsteede et al., 2003; Sanmartín et al., 2004), which contrasts with the findings in *M. chimango* from Chile, which featured prevalence rates of up to 70% (San Martín et al., 2006; Oyarzún-Ruiz et al., 2016). For the Harris’s hawk, there is a previous record of unidentified capillariid eggs from Brazil (Santos et al., 2015). Thus, the present record represents a new host-parasite association.

*Centrorhynchus* Lühe, 1911 has been reported worldwide in several species of diurnal and nocturnal birds of prey, including Accipitriformes, Falconiformes, and Strigiformes (Travassos, 1926; Yamaguti, 1963; Illescas Gomez et al., 1993; Sanmartín et al., 2004; Papazahariadou et al., 2008; Richardson & Kinsella, 2010; Santoro et al., 2010; Komorová et al., 2017). Species of this genus have an indirect life cycle with arthropods as intermediate hosts, and reptiles and amphibians as paratenic hosts (Atkinson et al., 2008). There are several species of *Centrorhynchus* recorded from South American countries, e.g. Argentina (Drago et al., 2015; Steinauer et al., 2019), Brazil and Ecuador (Travassos, 1926; Yamaguti, 1963; Melo et al., 2013), Nicaragua (Schmidt & Neiland, 1966), Paraguay (Smales, 2013), Colombia and Panama (Thatcher & Nickol, 1972), and Chile (Yamaguti, 1963; Grandón-Ojeda et al., 2018, 2019). For the Harris’ hawk, *Centrorhynchus virius* Smales, 2013 has been recorded from Paraguay (Smales, 2013). In the present study, the isolated acanthocephalan could not be identified to a specific level given its poor state of preservation.

*Cladotaenia* Cohn, 1901 is a tapeworm parasitizing wild birds as definitive hosts; meanwhile, micromammals act as intermediate hosts (Yamaguti, 1959). Some of the species recorded from birds of prey are *Cladotaenia accipitris* Yamaguti, 1935, *C. banghami* Crozier, 1946, *C. circi* Yamaguti, 1935, *C. cylindracea* (Bloch, 1782), *C. fania* Meggitt, 1933, *C. feuta* Meggitt, 1933, *C. foxi* McIntosh, 1940, *C. freani* Ortlepp, 1938, *C. globifera* (Batsch, 1786), *C. oklahomensis* Schmidt, 1940, *C. secunda* Meggitt, 1928, and *C. vulturi* Ortlepp, 1938 (Yamaguti, 1959; Sanmartín et al., 2004; Santoro et al., 2010; Komorová et al., 2017). Of these, *C*. *globifera* seems to be the most common species recorded for birds of prey in Europe (Sanmartín et al., 2004; Komorová et al., 2017). The low prevalence of tapeworms reported in the present study is supported by previous studies where these parasites were considered to be scarce (Illescas Gomez et al., 1993; Krone, 2000; Sanmartín et al., 2004; Papazahariadou et al., 2008; Komorová et al., 2017), although in some cases, the prevalence was high, as reported by Santoro et al. (2010) in Spain. The finding of this genus represents the second record in a Neotropical bird of prey (see Justo et al., 2017; Drago et al., 2021; Oyarzún-Ruiz & González-Acuña, 2021), with the first record identified in *F. sparverius* from central Chile (González-Acuña et al., 2011). There is no specific identification for this tapeworm in South America, a situation that could be overcome by well-preserved material and the use of molecular tools.

*Neodiplostomum* Railliet, 1919 is a cosmopolitan trematode that has been reported in raptors of orders Accipitriformes, Falconiformes, and Strigiformes (Dubois, 1937; Krone, 2000; Gibson et al., 2002; Sanmartín et al., 2004; Papazahariadou et al., 2008; Richardson & Kinsella, 2010). It has an indirect life cycle, with amphibians as intermediate hosts, and reptiles and mammals as paratenic hosts (Gibson et al., 2002). *Neodiplostomum travassosi* is a Neotropical species recorded from diurnal and nocturnal birds of prey, including toucans and cormorants as hosts (Dubois, 1937; Lunaschi & Drago, 2005). Raptor species acting as hosts for this fluke are *Buteogallus meridionalis* (Latham, 1790) (Accipitriformes), *Caracara plancus* (Miller, 1777) (Falconiformes), *Lophostrix cristata* Daudin, 1800, *Pulsatrix perspicillata* (Latham, 1790), *Athene cunicularia* (Molina, 1782) and *Strix* sp. (Strigiformes) from Argentina (Lunaschi & Drago, 2005; Drago et al., 2014, 2015) and Brazil (Dubois, 1937). Also, an unidentified *Neodiplostomum* was recorded for *B. magellanicus* in Chile by Grandón-Ojeda et al. (2018). The identity of this last taxon is required to establish whether it belongs to the species reported here. For the Harris’s hawk, there is a previous record of *Neodiplostomum biovatum* Dubois, 1937 from Brazil (Dubois, 1937). Thus, the present finding represents a new host-parasite association and marks the expansion of the geographical distribution of this species.

In the present study, a high proportion of birds – over 50% – were parasitized by helminth parasites, a situation that is aligned with the findings of previous studies of other species of raptors (Illescas Gomez et al., 1993; Krone, 2000; San Martín et al., 2006; Oyarzún-Ruiz et al., 2016; Komorová et al., 2017). In relation to the geographical expansion of parasites and the additional host-parasite associations reported here, the ectoparasites *C. nanum*, *N. chanabense*, and *Pseudalloptinus* sp., and helminths *P. alata*, *Microtetrameres* sp., *C*. (*H*.) *americana* and *N. travassosi* were recorded for the first time in Chile. Also, *C. nanum*, *N. chanabense*, and *C*. (*H*.) *americana* are recorded for the first time in Neotropical birds of prey. On the other hand, the specific status of *Craspedorrhynchus* sp., *Pseudalloptinus* sp., *Microtetrameres* sp., and *Cladotaenia* sp. is required to determine if they belong to new scientific taxa.

Most of the isolated helminths in the present study, e.g. *P. depressum*, *P. alata*, *Microtetrameres* sp., *C*. (*H*.) *americana*, *Centrorhynchus* sp., *N. travassosi*, and *Cladotaenia* sp., have indirect life cycles, using invertebrates such as earthworms and arthropods or rodents as intermediate hosts; others, such acanthocephalans, use amphibians and reptiles as paratenic hosts (Yamaguti, 1959; Anderson, 2000; Atkinson et al., 2008; Richardson & Kinsella, 2010). The Harris’s hawk is an opportunist, consuming not only medium and small mammals, reptiles, or birds, but also invertebrates (Pavez, 2019). Thus, this dietary behavior may explain the presence of a diversity of heteroxenous helminths reported here.

We did not consider coprological analyses; thus, additional surveys should consider conducting coprological analyses to evaluate the presence of protozoans such as *Eimeria* sp., *Caryospora*, *Cryptosporidium*, *Giardia*, *Sarcocystis*, and *Trichomonas*, among others, despite their scarce presence (see Krone, 2000; Papazahariadou et al., 2008; Santana-Sánchez et al., 2015; Santos et al., 2011).

Raptors seem to be tolerant to the presence of helminth parasites; however, under certain circumstances, such as stress, there could be severe health impairment on these hosts (Illescas Gomez et al., 1993; Krone, 2000; Krone & Cooper, 2002; Santoro et al., 2010; Komorová et al., 2017). Although some isolated helminths have reportedly been the cause of disease (e.g., *Cyathostoma*, *Cladotaenia*, and *Centrorhynchus*) with evident lesions (Lavoie et al., 1999; Krone, 2000; Atkinson et al., 2008; Santoro et al., 2010; Vaughan-Higgins et al., 2013), the birds necropsied in our study did not show any signs of pathological lesions associated with the parasites. This finding could have been associated with the low parasitic load recorded here. On the other hand, the highest parasitic load recorded was for *N. travassosi*. Although there are no data on the pathological consequences of this fluke species, these parasites are generally considered non-pathogenic for birds (Atkinson et al., 2008).

The inclusion of deposited specimens from a museum (MNHN) in this study demonstrated the utility of such collaboration, as two additional ectoparasitic species, which were not found on the necropsied birds nor those from CR, were collected from these samples. Thus, future studies on the parasites obtained from wild animals should include deposited specimens. However, caution must be considered because of the possibility of contamination with ectoparasites from other bird skins is possible.
